# Microvascular Complications in Type 1 Diabetes: A Comparative Analysis of Patients Treated with Autologous Nonmyeloablative Hematopoietic Stem-Cell Transplantation and Conventional Medical Therapy

**DOI:** 10.3389/fendo.2017.00331

**Published:** 2017-11-23

**Authors:** Jaquellyne G. Penaforte-Saboia, Renan M. Montenegro, Carlos E. Couri, Livia A. Batista, Ana Paula D. R. Montenegro, Virginia O. Fernandes, Hussain Akhtar, Carlos A. Negrato, Kelen Cristina Ribeiro Malmegrim, Daniela Aparecida Moraes, Juliana B. E. Dias, Belinda P. Simões, Marilia Brito Gomes, Maria Carolina Oliveira

**Affiliations:** ^1^Post Graduate Program in Medical Sciences, Federal University of Ceará, Ceará, Brazil; ^2^Federal University of Ceará, Ceará, Brazil; ^3^Center for Cell-Based Therapy, Regional Blood Center of Ribeirão Preto, Ribeirão Preto Medical School, University of São Paulo, Ribeirão Preto, Brazil; ^4^Department of Internal Medicine, Ribeirão Preto Medical School, University of São Paulo, Ribeirão Preto, Brazil; ^5^University of Oslo, UIO, Oslo, Noruega; ^6^Brazilian Society of Diabetes, São Paulo, Brazil; ^7^State University of Rio de Janeiro, Rio de Janeiro, Brazil

**Keywords:** type 1 diabetes, autologous nonmyeloablative hematopoietic stem-cell transplantation, residual B-cell function, glycemic control, microvascular complications

## Abstract

**Objective:**

To explore the impact on microvascular complications, long-term preservation of residual B-cell function and glycemic control of patients with type 1 diabetes treated with autologous nonmyeloablative hematopoietic stem-cell transplantation (AHST) compared with conventional medical therapy (CT).

**Research design and methods:**

Cross-sectional data of patients treated with AHST were compared with patients who received conventional therapy from the Brazilian Type 1 Diabetes Study Group, the largest multicenter observational study in type 1 diabetes mellitus in Brazil. Both groups of patients had diabetes for 8 years on average. An assessment comparison was made on the presence of microvascular complications, residual function of B cell, A1c, and insulin dose of the patients.

**Results:**

After a median of 8 years of diagnosis, none of the AHST-treated patients (*n* = 24) developed microvascular complications, while 21.5% (31/144) had at least one (*p* < 0.005) complication in the CT group (*n* = 144). Furthermore, no case of nephropathy was reported in the AHST group, while 13.8% of CT group (*p* < 0.005) developed nephropathy during the same period. With regard of residual B-cell function, the percentage of individuals with predicted higher C-peptide levels (IDAA1C ≤ 9) was about 10-fold higher in the AHST group compared with CT (75 vs. 8.3%) (*p* < 0.001) group. Among AHST patients, 54.1% (13/24) had the HbA1c < 7.0 compared with 13.1% in the CT (*p* < 0.001) group.

**Conclusion:**

Patients with newly diagnosed type 1 diabetes treated with AHST presented lower prevalence of microvascular complications, higher residual B-cell function, and better glycemic control compared with the CT group.

## Introduction

Type 1 diabetes mellitus (T1DM) is an autoimmune disease that results from autoreactive CD4^+^ and CD8^+^ lymphocytes attack against pancreatic beta cells (1), leading to symptomatic diabetes and lifelong insulin dependence (2). Islet autoimmunity is the first stage of the disease (3) and, at the time of clinical diagnosis, about 60–90% of the beta cells are destroyed or dysfunctional (1). Immunotherapy aims to preserve the remaining mass of B cells by attenuation of the activated, autoreactive T cells (4).

Bone marrow transplantation (BMT) has been studied as a therapeutic approach for T1DM since the 1980s (5). Initially, studies suggested that allogeneic BMT primarily for hematological disease could potentially transfer T1DM and in some instances, BMT could reverse it (6). Additionally, it was demonstrated that immunological intervention in newly diagnosed type 1 diabetic patients resulted in a slower decline of beta-cell function (7).

Between 2004 and 2010, Voltarelli et al. performed autologous nonmyeloablative hematopoietic stem-cell transplantation (AHST) in individuals with newly diagnosed T1DM (8–10). Twenty-one out of 25 patients became insulin-free during hospitalization for AHST. In June 2015, after a mean follow-up of 87 months, three were continuously insulin-free and 18 patients resumed exogenous insulin use after mean period of 33 months (ranging from 6 to 100 months). Mean area under the curve of C-peptide levels significantly increased from baseline compared with 6, 12, 24, 36, and 48 months post-AHST (10). At 60 and 72 months, most patients resumed insulin therapy and their C-peptide declined. Residual B-cell function was similar to that before transplant (10).

Several studies, including the Diabetes Control and Complications Trial (DCCT), demonstrated the association between high C-peptide levels and lower frequency of microvascular complications such as retinopathy, neuropathy, and diabetic nephropathy (11–13). For this reason, it was hypothesized that therapy with AHST could preserve B-cell function, improve metabolic control, and reduce microvascular complications.

Thus, the aim of this study was to compare the patients treated with AHST and those treated with the conventional medical therapy (CT) from the Brazilian Type 1 Diabetes Study Group (BrazDiab1). The following parameters were used in the comparison: the occurrence of microvascular complications, long-term preservation of residual B-cell function, and the patient’s glycemic control. This is the first analysis of the AHST through a comparator group for T1DM.

## Materials and Methods

### Study Design and Subjects

#### Hematopoietic Stem-Cell Transplantation (AHST) Group

Twenty-five patients with <6 weeks of diagnosis of t1dm aged between 13 and 31 years old were included in this group from January 2004 until May 2010 at the Center for Cell-based Therapy at the Ribeirão Preto Medical School—University of São Paulo, Brazil.

The rationale of AHST is based on the theory of “immunologic reset.” A complete study protocol was presented elsewhere (8–10). In brief, peripheral hematopoietic stem cells were mobilized with cyclophosphamide plus a granulocyte-colony stimulating factor. Stem cells were then harvested from the peripheral blood through apheresis, which was continued daily until the number of harvested progenitor cells reached a minimum of 3.0 × 10^6^ CD34^+^ cells/kg body weight. After approximately 15 days from beginning of mobilization, patients were conditioned with rabbit anti-thymoglobulin (ATG) for 5 days plus cyclophosphamide in the first 4 days. Intravenous infusion of autologous hematopoietic stem cells was performed on the sixth day with the aim of restoring immune balance. The AHST database was obtained from patient chart review in 2015. One patient lost the follow-up and was not included in this analysis.

#### The Conventional Therapy from Brazilian Type 1 Diabetes Study Group (CT)

The BrazDiab1 study group performed a multicenter retrospective, cross-sectional study on individuals with T1DM between December 2008 and December 2010, in 28 public clinics from the secondary and tertiary level of health care, from 20 cities (population > 100,000) from all the five Brazilian geographical regions (North, Northeast, Southeast, South and Midwest). All patients received health care from the National Brazilian Health Care System (NBHCS), and were regularly treated by an endocrinologist. All data were obtained from a single examination performed by trained medical professionals.

Details of BrazDiab1 have been published elsewhere (14). Briefly, participants were interviewed and demographic data were collected through a questionnaire. Furthermore, the following variables were assessed through an interview during a clinical visit and the patient’s medical records review.

#### Eligibility Criteria and Patient Selection

**Figure 1:**
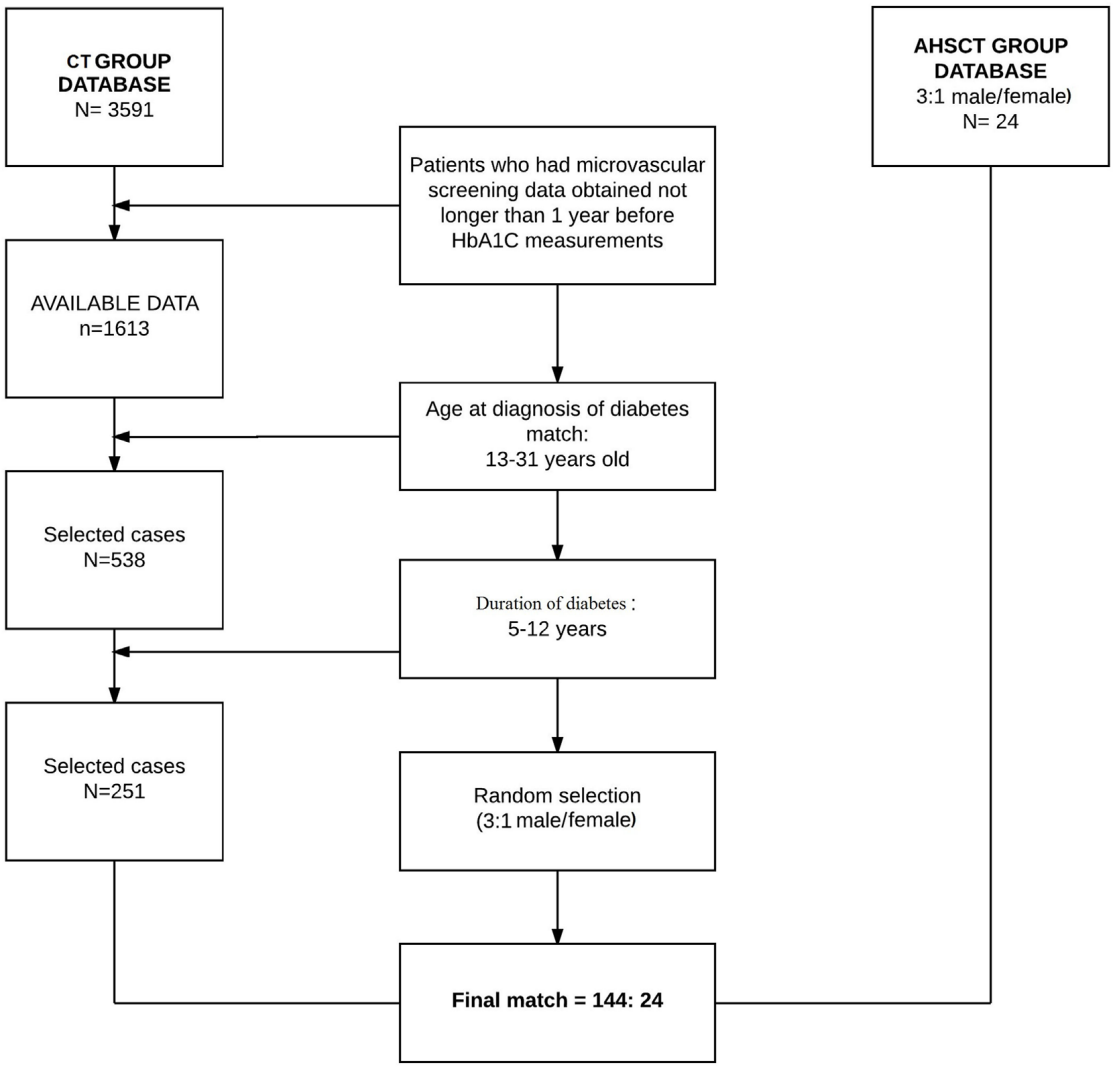
Case selection from both compared groups for data analysis.

Patients enrolled in the BrazDiab1 (*n* = 3,591) were primarily included. In the second step, the patients who were clinical screened for retinopathy, neuropathy, diabetic nephropathy, and HbA1c [National Glycohemoglobin Standardization Program method, including high-performance liquid chromatography (HPLC) and immunoturbidimetry] were selected. It was considered a 1-year timeline from the data collection (*n* = 1,613). Then, at the third stage of selection, the patients were matched for the age at diagnosis of diabetes (*n* = 538) and duration of diabetes (*n* = 251) to the AHST group. For the final stage, a matching of gender was made; BrazDiab1 women underwent a random selection through the SPSS program to maintain the approximate proportion of 30% of women present in the transplant group. A total of 144 patients were included in the final analysis (Figure 1).

#### Microvascular Complications

In both groups, diabetic retinopathy was screened by fundoscopy by a local ophthalmologist; nephropathy by microalbuminuria and glomerular filtration rate, defined by the Cockcroft–Gault equation (15); distal symmetric polyneuropathy was assessed by questioning the patients about symptoms of neuropathy, including paresthesia, dulled sensation, and pain in the feet and a neurological exam on the sensation of perception of vibration and 10-g monofilament pressure at the distal plantar of both great toes and metatarsal joints. The presence of microvascular complications was defined according to the American Diabetes Association Recommendations (16).

#### Residual Beta-Cell Function

The C-peptide values were measured only on the AHST group. Consequently, the residual B-cell function was evaluated indirectly in both groups using a model of insulin-dose adjusted HbA1c (IDAA1C), calculated as [A1C (%) + 4 × insulin dose (units per kilogram per 24 h)]. Values of IDAA1C ≤ 9 were associated with a predicted stimulated C-peptide (SCP) > 300 pmol/L and proposed as a way to evaluate clinically meaningful changes in intervention therapies that are aimed to preserve/regenerate B-cell function in new onset of T1DM (17). We followed the same conversion ratio to assess residual B-cell reserve.

#### Glycemic Control and Insulin Dose

Insulin-dose adjusted HbA1c < 7.0 (53 mmol/mol) was used as the marker for the long-term good metabolic control, according to the ADA recommendation (16). HbA1c was assessed using National Glycohemoglobin Standardization Program methods, including HPLC and immunoturbidimetry. Additionally, we also evaluated the percentage of patients from each treatment group with daily insulin doses <0.5 U/kg and HbA1C of <6.5% (48 mmol/mol) (4, 18).

#### Statistical Analysis

Continuous variables were presented as median and SD, and the categorical variables as absolute frequency and percentage. The comparative analysis between the two groups was estimated using the unpaired Student *t*-test or Mann–Whitney *U* test for continuous variables and the Pearson’s chi-square test or Fisher’s exact test for the categorical variables. Fisher’s exact test was used to compare the microvascular and glycemic control parameters of the analysis. The chi-square test was performed for the residual beta-cell function analysis and for insulin-dose comparison, Mann–Whitney test. Results were considered significant at *p* < 0.05.

## Results

### Patient Characteristics

Data of 24 subjects from AHST were contrasted against the data of 144 subjects from the CT (70% males in both, *p* = 0.945). The groups were well matched; in both, the age at diabetes diagnosis varied from 13 to 31 years, and the median was 16.0 years (p25–75: 15.5–20.5) for AHST group and 17.0 (p25–75: 14–22) for CT group (*p* = 0.964). Diabetes duration varied from 5 to 12 years for both groups, and the median was 9.0 years (p25–75: 7.5–10.0) for the AHST group and 8.0 years (p25–75: 6.0–10.0) for the CT group (*p* = 0.325).

Demographic characteristics of the study population are summarized in Table 1.

**Table 1:** Gender, t1dm duration and age range distribution for the AHST and CT groups.

Variable	AHST group	CT group	*p*-Value

	*N* = 24	*N* = 144	
Gender, M (%)	70.8	70.1	0.945
Duration of diabetes, years	9.0 (7.5–10.0)	8.0 (6.0–10.0)	0.325^a^	
Median (p25–75%)
Age at diagnosis, years	16.0 (15.5–20.5)	17 (14.0–22.0)	0.964^a^
Median (p25–75%)
13–16 years (%)	54.2	44.4	
17–21 years (%)	29.2	29.2	
22–33 years (%)	16.7	25.0	

*^a^Mann–Whitney test*.

*AHST, autologous nonmyeloablative hematopoietic stem-cell transplantation; CT, conventional therapy group from Brazilian Type 1 Diabetes Study Group*.

### Microvascular Complications

The AHST treatment group had lower prevalence of at least one microvascular complication. While none of the patients in the AHST group developed any microvascular complication, 21.5% (31/144) from CT group had at least one (*p* = 0.005). When microvascular complications were evaluated separately, the prevalence of nephropathy was significantly higher in the CT group (20 vs. no cases) (*p* = 0.037). Regarding neuropathy and retinopathy, there were nine (6.25%) and eight (5.5%) cases in the CT group vs. no cases in the AHST group (*p* = 0.28 and *p* = 0.24, respectively) (Table 2).

**Table 2:** Comparison of occurrence of diabetes microvascular complications and residual B-cell function in the AHST and CT groups.

Variable	AHST group	CT group	*p*-Value

	No./total	No./total	
**Microvascular outcomes**			
Any microvascular complication	0/24	37/144	0.005^a^
Diabetic nephropathy	0/24	20/144	0.037^a^
Diabetic retinopathy	0/24	8/144	0.283^a^
Diabetic neuropathy	0/24	9/144	0.241^a^
Residual B-cell function (IDAA1C ≤ 9.0)	%	%	
Total	75.0	8.3	*p* < 0.001^a^
<18 years old	66.6	5.4	*p* < 0.001^a^
≥18 years old	88.8	11.4	*p* < 0.001^a^

*^a^Fisher test*.

*IDAA1C, insulin-dose adjusted HbA1c; AHST, autologous nonmyeloablative hematopoietic stem-cell transplantation; CT, conventional therapy group from Brazilian Type 1 Diabetes Study Group*.

### Residual B-Cell Function

The AHST group showed a better residual B-cell function through the IDAA1C method. The percentage of patients with IDAA1C ≤ 9 was about 10-fold higher in the AHST group as compared with the CT group (75 vs. 8.3%; *p* < 0.001). Among patients <18 years old, 66.6% from AHST group vs. 5.4% from CT group were presented with IDAA1C ≤ 9 (*p* < 0.001); among patients ≥18 years old, the difference was found to be 88.8 vs. 11.4%, respectively (*p* < 0.001).

### Glycemic Control and Insulin Dose

The median dose of insulin was 65% lower among AHST group as compared with CT group (0.30 UI/kg/day vs. 0.85 UI/kg/day; *p* < 0.001). The median HbA1c values were significantly lower in AHST group as compared with CT group (6.99 vs. 9.32%; *p* < 0.001) despite the use of lower insulin doses. Among AHST patients, 54.1% (13/24) had HbA1c < 7.0 (53 mmol/mol) as compared with 13.1% in CT group (*p* < 0.001). In addition, at any given HbA1C intervals, patients in the AHST group achieved the intervals at lower insulin-dose cutoffs than in the conventional group (Table 2). The proportion of patients who presented with HbA1C of <6.5% (48 mmol/mol) and insulin dose <0.5 UI/kg/day was higher in AHST group than in conventional group—41.6 vs. 0.67% (*p* < 0.001). Only 33.3% of patients in the AHST group used rapid-acting and intermediate/long-acting insulins compared with 87.5% in CT group (*p* < 0.001). None and two patients, respectively, were on insulin pump therapy in the AHST and CT groups. Additionally, 12.5% (3/24) of patients from AHST group were not taking insulin compared with no cases in the CT group (*p* < 0.001). All these three patients had HbA1C of <7.0% (53 mmol/mol). In the year 2009, with a mean follow-up of 2.4years, 52.1% patients from AHST were insulin-free. Since 2008, every patient who stopped insulin use started to use sitagliptin 100 mg/day. A summary of the data about glycemic control, insulin use, and partial remission T1DM for both groups is described in Table 3.

**Table 3:** Glycemic control and insulin use for the AHST and CT groups.

**Patients characteristics**	**AHST group**		**CT group**		***p*-Value**
HbA1C (%)	6.9 (6.0–7.6)		8.7 (7.7–10.4)		<0.001^b^
Median (p25–75)
HbA1C (%) (mmol/mol)	52 (42–60)		72 (61–90)	
Median (p25–75)	
Insulin dose (UI/kg)	0.3 (0.23–0.35)		0.8 (0.7–0.9)		<0.001^b^	
Median (p25–75)
Insulin use, *n* (%)	21^a^ (87.5)		144 (100.0)		<0.001^b^
Only intermediate/long acting, *n* (%)	13 (54.1)		16 (11.1)		<0.001^b^
Intermediate/long acting plus short acting, *n* (%)	8 (33.3)		128 (88.8)		<0.001^b^
HbA1C < 6.5% (48 mmol/mol) with insulin dose <0.5 UI/kg, *n* (%)	10 (41.6)		1 (0.7)		<0.001^b^

**HbA1C intervals (% and mmol/mol)**	***n* (%)**	**Ins/kg/day**	***n* (%)**	**Ins/kg/day**	

≤6.99 (53)	13 (54.1)	0.29	19 (3.1)	0.68	<0.001^b^
7.0–7.99 (53–64)	8 (33.3)	0.31	25 (17.3)	1.01	
8.0–8.99 (64–75)	1 (4.1)	0.40	33 (22.9)	0.82	
>9.0 (75)	2 (8.3)	0.25	67 (46.5)	0.91	

*Glycemic control goal: Hba1c < 7.0%*.

*^a^The three patients who were not taking insulin presented with HbA1c < 7.0% and were using sitagliptin 100 mg/day*.

*^b^Mann–Whitney test*.

*AHST, autologous nonmyeloablative hematopoietic stem-cell transplantation; CT, conventional therapy group from Brazilian Type 1 Diabetes Study Group*.

## Discussion

Trials analyzing the effects of AHST in new-onset t1dm have shown promise in significantly attenuating the loss of insulin secretion (8, 9, 19, 20). The Voltarelli et al.’s publication was the first study to analyze the safety and efficacy of autologous stem-cell transplantation in humans. However, one of the recognized limitations of this study was the absence of a control group.

To the best of our knowledge, this is the first paper that compared late outcomes from patients submitted to AHST and subjects treated with conventional therapy, matched by gender, age at diagnosis of T1DM, and duration of disease.

According to literature, the prevalence of microvascular complications in T1DM varied considerably. However, it has been established that the strict control of glucose levels, especially in patients with a recent diagnosis of diabetes, and the maintenance of higher levels of C-peptide secretion result in a lower frequency of microvascular complications regardless of the insulin regimen (11–13). A recent Cochrane Library review showed that even in intensive glucose control group, the microvascular complication incidence was 6.2, 16.3, and 4.9% for retinopathy (follow-up: 5–6.5 years), nephropathy (follow-up: 3.5–6.5 years), and neuropathy (follow-up: 5–6.5 years), respectively (13), which is similar to our data in the group treated with conventional therapy. Another recent study conducted at the University of Poznan in subjects with new-onset type 1 diabetes demonstrated an association between the absence of partial remission of diabetes at any time in the follow-up (defined as Hba1C of <6.5% (48 mmol/mol) associated with insulin dose ≤0.3 UI/kg/day and stimulated C-peptide levels > 500 pmol/mL) and the incidence of microvascular complications. In the remitter group, at the end of 7 years, 7.6% of the patients had at least one microvascular complication, while in the non-remitter group the incidence was 46.4% (21). In contrast, none of the AHST patients after an average follow-up of 8.7 years presented any microvascular complications.

Based on the fact that the majority of patients resumed insulin use and C-peptide levels started do decline after 60 months after AHST (10), these data suggest that the effectiveness transplantation in reducing or delaying the development of microvascular complications is multifactorial. It is probably also dependent on mechanisms that go beyond the preservation of beta cells and improvement of glycemic control, and it should be considered in the remodeling of the diabetic microenvironment as proposed by Ahmed El-Badawy et al. (22).

The direct measurement of C-peptide has been recommended to provide the most appropriate primary outcome in trials evaluating the efficacy of therapies to preserve B-cell function (23). However, IDAA1C has been shown to be a good predictor of SCP levels, with a strong inverse correlation between SCP and IDAA1C, despite the fact that its substance did not fully predict C-peptide responses (24). It was evaluated initially in children and adolescents with T1DM (17). According to this model, an IDAA1C ≤ 9 corresponds to a predicted level of SCP > 300 pmol/L (17); however, an IDDAC ≤ 9 can significantly decrease the number of subjects with SCP ≥ 200 pmol/L (25). Additionally, Buckingham et al. in evaluating the patients at aged between 7 and 45 years old with a recent diagnosis of t1dm found that virtually all participants with IDAA1C < 9 retained substantial insulin secretion, with a peak of C-peptide levels > 200 pmol/L (26).

The same group mentioned above, who initially described the IDAA1c (17), also carried out a study in 129 Danish children and adolescents. They have confirmed the findings of the first trial, which established the relationship between IDAA1C ≤ 9 and partial remission of T1DM (SCP > 300 pmol/L). Although IDAA1C ≤ 9 has a good sensitivity in predicting an SCP > 300 pmol/L, the specificity was reduced, with 34 patients presented a level of SCP > 300 pmol/L and IDAA1C > 9 at 12 months. Most of them were >10 years old and presented poor glycemic control. Thus, the higher C-peptide levels in this group may not reflect a lower severity of T1DM, but, in part, may be a consequence of reduced insulin sensitivity during puberty and use of mixed meal with more carbohydrates in the Danish cohort than in the initial cohort. Ultimately, the authors were able to prove the prognostic and diagnostic power of the IDAA1c measure that presents a good estimate of final insulin production, and thus it can be used in interventional studies aimed at reducing the progression of t1dm and preserving beta-cell function (27).

Analysis of natural history of residual insulin secretion in T1DM showed that the level of SCP continued to diminish over time, and at the end of 4 years, regardless of age, only a small percentage of subjects maintain stable B-cell function. In this period, only 2.7 and 20.4% of the patients aged between 12 and 17 and ≥18 years, respectively, maintained IDAA1C ≤ 9 (25). These results correspond well with our conventional therapy group after an average follow-up of 8.2 years. In contrast, in AHST group the prevalence of IDAA1C ≤ 9 was 66.6 and 88.8% for respective age groups. Another trial evaluating 3,657 patients with T1DM (below 18 years old at diagnosis) found that in only 5% of the IDAA1c was ≤9 after 6 years of diagnosis (24). Thus, despite the limitations of a cross-sectional design derived from a retrospective cohort, our data demonstrated that the therapy with AHST has the potential for improving long-term beta-cell function.

Specific mechanisms of how AHST improves B-cell function are yet not fully understood. This is due to attaining the immunological tolerance, hematopoietic stem-cell infusion, or both (28). The analysis of the immunological status after AHST showed a newly regenerated immune system that is more tolerant to auto-antigens, with an increase in thymic production of naïve T-cell populations, a normalization of the T-cell receptor repertoire, and an increase in the population of T-regulatory cells (Tregs) (10, 29). Tregs promote self-tolerance through the modulation of activated effector cells during antigen reexperiencing after transplantation (30) and they may also inhibit spontaneous autoreactive proliferation in lymphopenic environments (31). They increased after AHST was associated with prolonged remission of disease. In addition, lower autoreactive islet-specific CD8^+^ T-cell (aCTL) frequencies were also associated with longer insulin-free periods. However, AHSC was not able to affect specific islet autoreactivity, i.e., with no significant changes in a CTL frequencies following transplantation (10).

The main therapeutic mechanism of AHST seems to be the immunological resetting. However, stem cells may have therapeutic potential associated with their intrinsic regenerative capacity as well as the immunomodulatory potential. The immunomodulatory properties could arrest beta-cell destruction, preserve residual beta-cell function, facilitate endogenous beta-cell regeneration, and prevent the recurrence of autoimmunity. The regenerative capacity could promote a self-replenishing supply of glucose-responsive insulin-secretin cells for transplantation (32–34).

However, the ability of transplanted hematopoietic cells to directly differentiate into pancreatic beta cells has not yet been clearly demonstrated. While human stem-cell embryos are capable of differentiating and expressing insulin-producing genes, rat stem-cell embryos are able to generate cells of the pancreatic islets through genetic modification (35, 36).

The general goal of insulin therapy in t1dm is to reduce hemoglobin A1C (A1C) to ≤7.0% (53 mmol/mol) (16). However, this better glycemic control may be accompanied by undesirable effects, such as increased episodes of hypoglycemia (13) need for larger and more frequent doses of insulin. In clinical practice, it has been observed that only a minority of diabetic patients, irrespective of types of insulin or dosing schedules, can maintain HbA1c goal. In a recent publication, Baxter et al. in evaluating a representative cohort of adults with t1dm in the United Kingdom health system found that, despite the use of human insulin, insulin analogs, basal-bolus insulin, and an increasing use of insulin pumps <30% patients with t1dm have HbA1c levels <7.5% (59 mmol/mol) (37).

Similarly, a Brazilian population-based study observed that only 11.6% of the patients presented with HbA1c < 7.0% (53 mmol/mol) (14), similar to that observed in our population in the standard treated group. Garg et al. in a very recent randomized, multicenter trial evaluating the use of sotagliflozin—a new oral inhibitor of sodium–glucose cotransporters 1 and 2—in patients with T1DM showed that the association of this drug with existing insulin regimens was associated with a significant reduction in glycated hemoglobin values and in daily insulin dose. However, at the end of 24 weeks, even among patients in the sotagliflozin group, only 29.6% had HbA1C < 7.0%, and the placebo-corrected reductions from baseline in the mean daily total insulin dose was 9.7% (38). In contrast, the majority of patients in the AHST group (54.1%) presented with HbA1C < 7.0% (53 mmol/mol) and the median dose of insulin was <65% in AHST group as compared with CT group.

Patients in the AHST group were presented with lesser use of rapid insulin and with lower values of HbA1C (*p* < 0.001) as compared with CT group. In addition, 12.5% (3/24) of patients in the AHST group did not need to take insulin; none of such patients were observed in the group treated with standard therapy. Furthermore, a study evaluating teplizumab in recently diagnosed individuals, with mean age of 19 years, just 5% (19/415) of the patients were insulin-free after a shorter period of 1 year (4). However, it should be mentioned that the percentage of insulin-free patients presented decreased with a larger follow-up in AHST group. Previous published data involving median follow-up of 2.4 years, relative to 23 of the 24 patients from AHST group, showed that 52.1% were free (8), as compared with 12.5% found here. This effect occurred in parallel with the reduction of beta-cell function over time. Nevertheless, mounting evidence is observed that AHST therapy appears to be able to achieve better glycemic control with a remarkable decrease needed for insulin administration.

In conclusion, AHST seems to induce a reduction in the prevalence of microvascular complications/diabetic nephropathy as compared with the conventional therapy group. Additionally, our data suggest enhanced preservation of the residual beta-cell function and an improvement in the glycemic control with lesser or no use of insulin.

However, important limitations of this study include a retrospective design, small number of patients, and indirect measurement of C-peptide levels. Another limiting factor is a relatively short duration of diabetes for the analysis of microvascular complications. Joint efforts should be maintained for the discovery of new therapeutic strategies for this disease including its long-term effect. The data presented here have important implications for t1dm treatment in Brazil and potentially elsewhere in the world.

## Ethics Statement

This study was carried out in accordance with the recommendations of Declaration of Helsinki with written informed consent from all subjects. The protocol was approved by the University of Ceara Hospital Research Ethics Board (Protocol no. 1886743).

## Author Contributions

JS contributed to the analysis of data and writing of the manuscript. CC, KM, DM, JD, BS, and MG contributed to the analysis of data. RM, LB, AM, VF, AH, and CN contributed to the writing, reviewing, and editing of the manuscript.

## Conflict of Interest Statement

The authors declare that the research was conducted in the absence of any commercial or financial relationships that could be construed as a potential conflict of interest.
